# Conservative Management of a Cervical Horizontal Root Fracture in a Mature Permanent Incisor: A Case Report

**DOI:** 10.1155/crid/6139604

**Published:** 2026-11-15

**Authors:** Giselle Anahí Zacharczuk, Pablo Alejandro Rodriguez

**Affiliations:** ^1^ Universidad de Buenos Aires. Facultad de Odontología Cátedra de Odontología Integral Niños Buenos Aires Argentina

**Keywords:** case report, conservative treatment, root fractures, splinting, traumatic dental injuries

## Abstract

Cervical root fractures in permanent teeth are uncommon traumatic injuries that may present significant therapeutic challenges and are associated with a poor prognosis. This report describes the conservative management of a cervical horizontal root fracture in a permanent maxillary central incisor in a healthy 13‐year‐old girl who presented 48 hours after a sports‐related trauma. Clinical examination revealed displacement and increased mobility of Tooth 21 and gingival bleeding. Periapical radiography showed a complete horizontal root fracture in the cervical third, at the level of the alveolar crest, with approximately 2 mm displacement between the fragments. The coronal fragment was repositioned and stabilized with a flexible splint fabricated from monofilament nylon fishing line and composite resin. A removable protective appliance was also provided during the initial 4 weeks to prevent premature contact and improve comfort. Considering the unfavorable prognostic factors, splinting was maintained for 7 months. At that time, localized gingival inflammation was observed around the splint, and the splint was removed. The gingival condition resolved within 1 week. Follow‐up continued, and a mouthguard was subsequently provided for use during sports activities. At the 12‐month follow‐up, the tooth remained asymptomatic and functional, with maintained pulp sensibility and findings compatible with favorable healing. The natural tooth was preserved.

## 1. Introduction

Most traumatic dental injuries (TDIs) occur in children and adolescents and may have long‐term functional, aesthetic, and psychosocial consequences [1]. Therefore, preservation of the affected teeth is a primary treatment goal, particularly in growing patients with developing dentition [2–5].

Root fractures are uncommon, accounting for approximately 0.5% to 7% of TDIs involving the permanent dentition [6]. They occur predominantly in maxillary central incisors and are most frequently observed in males between 11 and 20 years of age [6–8]. Horizontal and oblique root fractures divide the root into coronal and apical fragments. In most cases, the apical fragment remains stable within the alveolus, whereas the coronal fragment is displaced and mobile [1, 9, 10].

Diagnosis requires radiographic assessment, as clinical findings may resemble those of luxation injuries. Periapical radiographs obtained at different angulations may be necessary to identify the fracture line and determine its location and degree of displacement. Cone‐beam computed tomography (CBCT) may provide additional three‐dimensional information when conventional radiography is insufficient [1, 7, 11].

Fracture location is an important prognostic factor. Although apical and middle‐third fractures generally have more favorable outcomes, cervical root fractures present a greater therapeutic challenge and are associated with a less predictable prognosis [9, 12, 13]. According to the International Association of Dental Traumatology (IADT) guidelines, emergency management primarily consists of repositioning the displaced coronal fragment and stabilizing it with a passive flexible splint.

Although 4 weeks of stabilization is recommended for most root fractures, cervical root fractures require prolonged splinting because of the unfavorable fracture location and increased mobility of the coronal fragment, with stabilization for up to 4 months recommended in these cases [1, 3]. According to Cvek et al. [12], the duration of splinting may be determined individually based on the severity of the injury and findings at follow‐up radiographic examinations. Regular clinical and radiographic follow‐up is recommended to assess pulp status, detect complications, and monitor the healing process [1, 6, 8].

When a cervical fracture is located close to the gingival margin or alveolar crest, preservation of the coronal fragment may be particularly difficult. Extraction of the coronal fragment followed by orthodontic or surgical extrusion of the remaining root has therefore been proposed as an alternative treatment [9–14]. However, conservative management aimed at preserving the coronal fragment may be considered when favorable clinical conditions, adequate oral hygiene, and patient compliance are present [6, 8, 10].

The literature on conservative treatment of cervical root fractures remains limited, and the reported outcomes are heterogeneous [12]. Although preservation of the coronal fragment has been described, the management of fractures located at or near the alveolar crest remains clinically challenging, particularly when treatment is delayed and the coronal fragment is significantly displaced. This report describes the conservative management of a cervical horizontal root fracture in a permanent maxillary central incisor in an adolescent patient who presented 48 hours after trauma. The case is of particular clinical interest because preservation of the coronal fragment was pursued despite the cervical fracture location, significant fragment displacement, and delayed presentation, with prolonged stabilization and temporary occlusal protection, followed by clinical and radiographic monitoring of pulp sensibility, mobility, and radiographic changes at the fracture site over a 12‐month period.

## 2. Case Presentation

### 2.1. Patient Information

A 13‐year‐old female patient with no relevant medical history presented to the Department of Comprehensive Pediatric Dentistry, School of Dentistry, University of Buenos Aires, accompanied by her mother, 2 days after sustaining a dental traumatic injury while playing sports at a local club. The injury resulted from a direct impact of a hockey stick to the left maxillary central incisor while the patient was not wearing a mouthguard.

No previous dental trauma involving the maxillary anterior teeth was reported, and Tooth 21 had no known history of previous dental treatment or pathology before the injury. No relevant oral habits were reported.

Immediately after the injury, the patient was taken to the emergency department of a local hospital, where no definitive dental treatment was provided. Management was limited to obtaining a panoramic radiograph and prescribing amoxicillin 500 mg and ibuprofen 400 mg every 8 hours.

### 2.2. Clinical and Radiographic Findings

Clinical examination revealed contusion of the upper lip and gingiva in relation to Tooth 21. The crown was displaced in an incisal and palatal direction and exhibited Grade 2 mobility. Gingival sulcular bleeding was observed, and a fracture line was visible beneath the gingival sulcus on the buccal surface. (Figure 1a). The displaced coronal fragment caused premature contact with the opposing tooth, preventing proper occlusion and impairing masticatory function.

**Figure 1:**
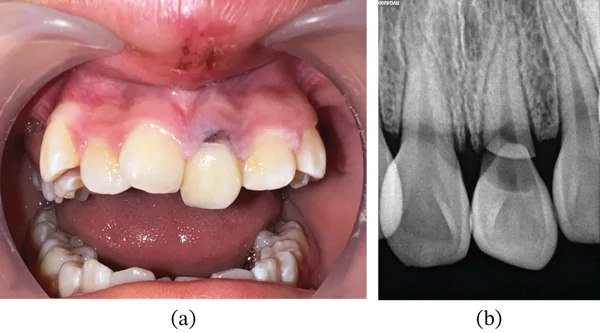
Initial clinical and radiographic examination: (a) Extrusive and lateral displacement of the crown of the left maxillary central incisor, with bleeding from the gingival sulcus and contusion of the upper lip. (b) Periapical radiograph showing a complete horizontal root fracture located in the cervical third of Tooth 21, complete root development, and no signs of periapical pathology.

The patient reported pain and difficulty eating because of the displacement and mobility of the injured tooth. Pulp sensibility and percussion tests were not performed in 21 at the initial examination because the patient was experiencing significant pain and was highly distressed.

Periapical radiograph showed a complete horizontal root fracture located in the cervical third of Tooth 21, at the level of the alveolar crest, with approximately 2 mm of displacement between the fragments. The tooth had complete root development, with no signs of periapical pathology (Figure 1b).

General dental examination revealed severe maxillary and mandibular crowding, with no carious lesions. Clinical and radiographic assessment identified no additional traumatic injuries in the adjacent teeth, which showed no mobility or tenderness to percussion or palpation.

### 2.3. Diagnostic Assessment

The definitive diagnosis was a complete horizontal root fracture in the cervical third of Tooth 21, at the level of the alveolar crest, with approximately 2 mm of displacement and mobility of the coronal fragment. Although displacement and mobility may also occur in luxation injuries, visualization of a fracture line beneath the gingival sulcus raised suspicion of a root fracture, which was subsequently confirmed by radiographic examination. No clinical or radiographic evidence of an associated alveolar bone fracture or additional traumatic injury to the adjacent teeth was identified.

A single initial periapical radiograph clearly demonstrated the horizontal root fracture and the displacement between the fragments. Although additional periapical projections at different angulations may be useful when a root fracture is not clearly visible, they were not considered necessary in this case because the fracture was clearly identified on the initial periapical image. A second periapical radiograph was obtained immediately after repositioning of the coronal fragment and placement of the flexible splint to document the posttreatment position.

A limited‐field CBCT examination (5 × 5 cm; voxel size, 0.1 mm) was also performed to further characterize the injury. It confirmed the presence of a single horizontal fracture in the cervical third of the root and showed the relationship between the fragments and the surrounding alveolar structures, with no evidence of an associated alveolar fracture or injury to adjacent structures.

### 2.4. Therapeutic Intervention

Considering the cervical location of the fracture, the dislocation and degree of displacement of the coronal fragment, and the time elapsed between the injury and presentation, the patient and her mother were informed that the affected tooth had a guarded prognosis. After obtaining the patient′s assent and her mother′s written informed consent, emergency treatment was performed (Table 1).

**Table 1:** Timeline of historical and current case information.

Time	Event
Day 0	Dental trauma during a field hockey match (without a mouthguard). Initial emergency care at another healthcare center. Panoramic radiograph obtained; amoxicillin 500 mg and ibuprofen 400 mg every 8 hours were prescribed.
48 hours after trauma	Presentation to the Department of Pediatric Dentistry, School of Dentistry, University of Buenos Aires. Clinical and radiographic examination revealed a horizontal cervical root fracture of the maxillary left central incisor with displacement of the coronal fragment.
Same visit	Coronal fragment repositioning by digital manipulation under local anesthesia. Flexible splinting using monofilament nylon fishing line and composite resin. A 0.08‐inch rigid removable thermoformed appliance was also provided to prevent premature occlusal contact and improve comfort. It was worn continuously except during toothbrushing and oral hygiene. Postoperative instructions included a soft diet, suspension of sports activities, and 0.12% chlorhexidine mouth rinse twice daily.
Same visit	Field CBCT performed to further characterize the root fracture and assess the relationship between the fractured fragments and surrounding structures.
1 week	Clinical follow‐up: Tooth 21 was asymptomatic, correct position of the coronal fragment maintained, and healthy gingival tissues.
4 weeks	Decreased mobility 21, increased comfort.
	Discontinue of removable appliance.
8 weeks	Clinical‐radiographic follow‐up 21: Favorable findings, with no clinical or radiographic signs of pathology. Tooth 21 responded positively to cold sensibility testing.
4 months	Continued favorable clinical and radiographic findings 21.
	Decision of continued splinting.
5–6 months	Monthly clinical monitoring during the extended stabilization period, with no clinically relevant changes observed.
7 months	Favorable clinical stability of Tooth 21. Enlargement of the interdental papillae and bleeding on probing were observed adjacent to the splint. The flexible splint was removed, and oral hygiene measures were reinforced.
7 months +1 week	Improvement of gingival condition.
	Custom‐made mouthguard.
12 months	No clinical or radiographic signs of pathology.
	Tooth 21: Positive response to cold sensibility testing, normal mobility, and normal response to percussion. CBCT demonstrated approximation of the fractured fragments and slight narrowing of the pulp space. Findings were considered compatible with favorable healing.
Future follow‐up	Annual clinical and radiographic examinations are planned for a minimum of 5 years.

Local infiltrative anesthesia was administered in the labial vestibule, and the coronal fragment was repositioned by applying firm digital pressure (Figure 2a). A flexible splint made of nylon fishing line was then bonded to the labial surfaces of Teeth 12, 11, 21, 22, and 23 using composite resin (Figure 2b). A periapical radiograph was subsequently obtained to confirm proper repositioning of the coronal fragment (Figure 2c).

**Figure 2:**
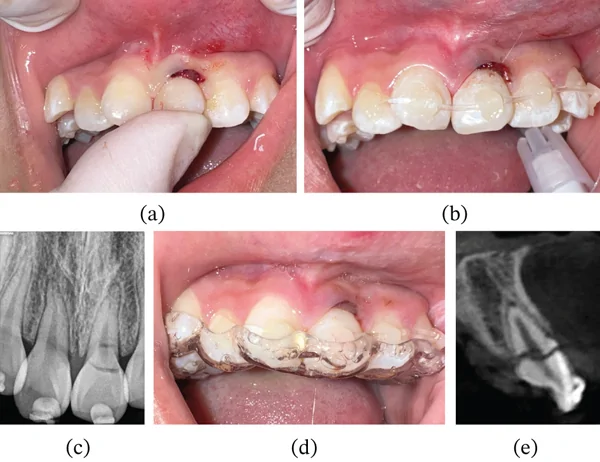
Treatment: (a) Manual repositioning of the coronal fragment by applying firm digital pressure. (b) Placement of a flexible splint fabricated with monofilament nylon fishing line and composite resin. (c) Postoperative periapical radiograph confirming correct repositioning of the coronal fragment. (d) Additional stabilization with a removable thermoformed occlusal splint. (e) Sagittal CBCT reconstruction shows the complete horizontal root fracture of Tooth 21 at the level of the alveolar bone crest.

During mandibular movements, premature contact between Tooth 21 and the mandibular central incisor was detected. Therefore, an alginate impression was obtained and a 0.08‐inch‐thick rigid thermoformed splint was fabricated as an adjunct to the flexible splint to minimize occlusal loading on the injured tooth, prevent repetitive contact, and improve patient comfort. Its fitness and ease of insertion and removal were verified. The patient was instructed to wear the appliance continuously, removing it only twice daily for cleaning the appliance and properly brushing her teeth (Figure 2d).

Besides, the patient was advised to avoid sports activities, follow a soft diet, and rinse twice daily with 0.12% chlorhexidine gluconate for 1 week. The importance of maintaining good oral hygiene was emphasized to minimize plaque accumulation and reduce the risk of local complications. Antibiotics prescribed at the time of injury were discontinued.

A CBCT was requested to allow a more accurate three‐dimensional assessment of the fracture and surrounding structures. Given the patient′s age and the need to minimize radiation exposure, a small field of view (5 × 5 cm) and a voxel size of 0.1 mm were selected. The scan confirmed the presence of a single complete horizontal root fracture located in the cervical third, at the level of the alveolar bone crest. It also confirmed correct repositioning of the coronal fragment and the integrity of the alveolar cortical plate and adjacent structures (Figure 2e).

### 2.5. Follow‐Up and Outcomes

During the first month, the patient attended weekly follow‐up visits. At the 1‐week follow‐up examination, she reported no pain, and clinical examination showed favorable healing of the soft tissues, with no signs of inflammation. Oral hygiene was adequate (Figure 3a).

**Figure 3:**
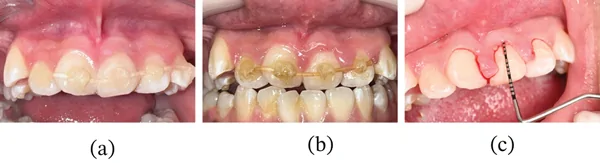
Follow‐up: (a) Clinical examination at 1 week showing favorable soft tissue healing with no signs of gingival inflammation. (b) Clinical appearance at 7 months showing inflammatory gingival changes. (c) Remotion of splint at 7 months.

At 4‐week follow‐up visit, clinical examination showed decreased mobility of Tooth 21. The patient also reported more comfort and increased confidence during mastication compared with the initial presentation. Based on these findings, the removable appliance was discontinued, whereas the flexible splint was maintained.

At the 8‐week follow‐up visit, Tooth 21 showed favorable clinical and radiographic findings, with no signs of clinical or radiographic pathology. The tooth responded positively to cold sensibility testing using refrigerant spray (Ice, Klepp) applied to the tooth on a large cotton pellet.

At the 4‐month follow‐up visit, although the recommended stabilization period had been completed, the initial displacement of the coronal fragment and the cervical location of the fracture led to the decision to continue flexible splinting for an additional 3 months, with close clinical monitoring. The patient was subsequently assessed monthly at 5 and 6 months, with no clinically relevant changes observed. This decision was individualized to the clinical circumstances of the case and was not based on a predefined protocol.

At the 7‐month follow‐up visit, inflammatory gingival changes associated with prolonged splinting were observed, including enlargement of the interdental papillae adjacent to the splint and bleeding on probing (Figure 3b). Given the favorable clinical stability of the tooth and these treatment‐associated periodontal findings, the splint was removed (Figure 3c). Following splint removal, Tooth 21 exhibited physiological mobility, a normal response to percussion, and a slightly increased response to cold sensibility testing compared with the adjacent teeth. The patient was instructed to improve her toothbrushing technique and increase the frequency of oral hygiene measures. The gingival condition had considerably improved 1 week after splint removal, although oral hygiene continued to be monitored.

At the 12‐month follow‐up visit, Tooth 21 remained asymptomatic, with no clinical or radiographic signs of pathology (Figure 4a,b). It responded positively to cold sensibility testing, exhibited physiological mobility, and showed a normal response to percussion (Figure 4c). Follow‐up CBCT demonstrated approximation of the fractured fragments and slight narrowing of the pulp space (Figure 4d). These findings were considered compatible with favorable healing. The periodontal tissues appeared clinically and radiographically healthy.

**Figure 4:**
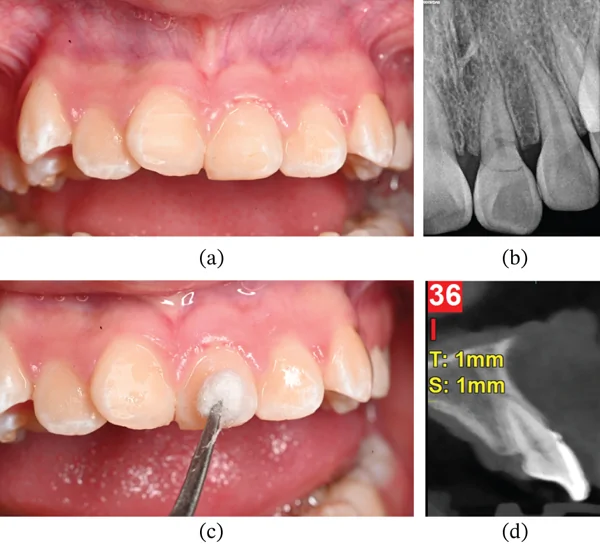
12 month follow‐up: (a) Clinical appearance at 12 months. (b) Radiographic findings compatible with favorable healing. (c) Positive response to cold sensibility testing. (d) CBCT at 12 months. The fracture site at the cervical level can be traced along and narrowing of pulp space can be seen.

The patient and her family were informed of the need for annual clinical and radiographic follow‐up examinations for a minimum period of 5 years. A new custom‐made mouthguard was fabricated, and they were advised of the importance of using it during sports activities to help prevent future TDIs.

## 3. Discussion

A root fracture is a significant TDI of considerable clinical complexity that requires the clinician to have a thorough understanding of its etiological mechanisms, the biological responses of the affected tissues, the available treatment options according to the type and location of the fracture, and the factors influencing prognosis [9].

In the present case, several factors were associated with poor prognosis. Among these, the location of the fracture was considered the greatest concern because of its proximity to the gingival margin and the biological and mechanical challenges inherent to this type of injury.

Cervical third root fractures are associated with a substantially less favorable prognosis than fractures located in the apical or middle third of the root because the coronal fragment is shorter and more mobile, reducing the likelihood of healing with hard tissue [12]. According to Abbott [9], the lack of bony support, limited periodontal attachment of the coronal fragment, and the risk of bacterial contamination of the fracture line substantially reduce the likelihood of healing, particularly when the fracture is located supracrestally in relation to the alveolar bone. In such cases, the author recommends immediate removal of the coronal fragment followed by restoration of the remaining root when feasible, or extraction if the tooth is considered nonrestorable [9, 15].

In a retrospective study of 534 permanent incisors with intra‐alveolar root fractures in patients aged 7–17 years, Cvek et al. [16] identified interposition of soft tissue between the fractured fragments as the main risk factor for tooth loss while reporting healing in approximately 30% of cervical fractures. Based on these findings, the authors considered a conservative treatment approach worthwhile and advisable before resorting to more arduous treatment alternatives.

In accordance with this conservative approach, the current IADT guidelines recommend repositioning the displaced coronal fragment, followed by stabilization with a flexible splint during the emergency visit, rather than immediate removal of the coronal fragment [1]. This recommendation formed the basis for the treatment strategy adopted in the present case. If the clinical outcome were unfavorable and the coronal fragment was ultimately lost, more complex restorative procedures might be required to re‐establish function and aesthetics. These may include orthodontic or surgical extrusion of the remaining root fragment to expose sufficient sound tooth structure for subsequent restoration [14].

In addition to the fracture location, the incisal and palatal dislocation and the 2 mm displacement of the coronal fragment were of particular concern, as dislocation and degree of displacement of the coronal fragment are important prognostic factors in teeth with root fractures. Healing, particularly healing with hard tissue, occurs significantly more frequently in teeth with an undislocated than in those with a dislocated coronal fragment [12]. Displacement may also result in varying degrees of pulpal injury, ranging from stretching of the neurovascular bundle to its complete severance, potentially leading to pulp necrosis. However, according to Abbott, under favorable conditions, revascularization of the pulp tissue may occur, promoting pulp survival and contributing to the healing process [9].

Another unfavorable aspect was the 48‐hour delay between the injury and initiation of treatment. Although repositioning of the coronal fragment should ideally be performed as soon as possible after the injury [10], available evidence suggests that a short treatment delay of a few days does not necessarily result in impaired healing, as healing of root fractures is influenced by multiple biological and mechanical factors [17]. Favorable healing may still occur, even in cases initially considered to have a poor prognosis, provided that accurate repositioning and adequate stabilization of the fractured fragments are achieved [8, 17].

The antibiotic therapy initiated at the first healthcare center was discontinued upon presentation to our institution, as current evidence does not support the routine use of systemic antibiotics for uncomplicated root fractures in the absence of indications of infection or associated soft tissue or systemic involvement [1, 17].

Regarding splinting, its primary purpose is to prevent (re)luxation of the repositioned coronal fragment without interfering with the healing process [18], ideally until a hard tissue callus has formed [6]. Although some studies have reported that splint type does not influence either the frequency or the type of healing [12], current dental traumatology guidelines recommend the use of flexible (physiological) splints rather than rigid splints, as they allow a certain degree of functional mobility while providing adequate stabilization [17].

In this case, the injured tooth was stabilized with a flexible splint fabricated from monofilament nylon fishing line bonded to the buccal face of teeth with composite resin. Nylon fishing line represents an alternative to conventional wire‐composite splinting systems, as it provides stabilization while preserving physiological tooth mobility. In addition, it is inexpensive, easy to place and remove, atraumatic, relatively aesthetic, hygienic, durable, and comfortable for the patient [19].

An additional removable appliance was provided primarily to prevent premature contact during certain mandibular movements and to improve the patient′s comfort. At the initial stage, the patient was highly apprehensive and concerned about losing the tooth while eating. The appliance contacted Tooth 21 and, given its rigid design, may also have provided an additional stabilizing effect during the initial healing period. It was not cemented, allowing the patient to easily insert and remove it, and was worn continuously except during toothbrushing and oral hygiene. Its use was discontinued after 4 weeks, whereas the flexible bonded splint was maintained.

Although flexible splinting of cervical root fractures is generally recommended for 4 months [1, 3], longer periods of stabilization have also been described in individual case reports [20–22]. Permanent fixation of the coronal fragment to adjacent noninjured teeth using a lingual orthodontic retention wire has also been reported [6]. In the present case, at the 4‐month follow‐up examination, the decision was made to extend flexible splinting for an additional 3 months because of the cervical location of the fracture and the initial displacement of the coronal fragment. The patient was monitored monthly during this extended period, with no clinically relevant changes observed at 5 and 6 months. At 7 months, favorable clinical stability was observed; however, inflammatory gingival changes associated with prolonged splinting prompted removal of the splint.

The decision to extend stabilization was individualized to the clinical circumstances of this case and should not be interpreted as evidence that prolonged splinting was responsible for the favorable outcome. A recent Bayesian network meta‐analysis found no significant differences in healing outcomes among different splint types for horizontal root fractures, including comparisons with no fixation, whereas fragment displacement was identified as an important factor potentially influencing healing [18]. However, the meta‐analysis included only six observational studies, and the authors noted that the available evidence remains limited [18]. In addition, the rigid thermoformed appliance used during the first 4 weeks may have provided an additional stabilizing effect, although its primary purpose was to control occlusal loading and improve patient comfort. Therefore, the relative contribution of the flexible splint, the thermoformed appliance, and the initial reduction of the displaced fragment to the observed outcome cannot be determined from this single case.

At 7 months, however, inflammatory gingival changes were observed, including enlargement of the interdental papillae adjacent to the splint and bleeding on probing. These findings may have been related to bacterial plaque accumulation around the nylon fishing line during the prolonged period [23]. Given the favorable clinical stability of Tooth 21, the splint was removed, and oral hygiene measures were reinforced. Following these measures, the gingival condition resolved within 1 week, supporting a temporal association between removal of the splint and improvement of the periodontal findings.

A mouthguard was fabricated for use during sports activities, and the patient was informed of the potential risk that any future traumatic injury could pose to the long‐term survival of the tooth [9].

Although the follow‐up is currently limited to 12 months, the clinical and radiographic outcome has been positive to date, with findings consistent with favorable healing. The tooth showed a positive response to cold sensibility testing with refrigerant spray. Narrowing of the pulp space was observed, which is a common finding following root fractures [1]. Even in cases of complete pulp space obliteration, this finding has not been associated with an adverse effect on tooth survival [16]. To date, no endodontic intervention has been required.

The patient reported being highly satisfied with the outcome, which is also relevant to the clinical management of a traumatic injury in an adolescent. Preservation of a maxillary central incisor may have important functional and psychosocial implications. The patient and her family were informed of the need for annual clinical and radiographic follow‐up examinations for a minimum period of 5 years [1].

Similar favorable outcomes following the conservative management of cervical root fractures have also been reported in other case reports [20–22, 24]. Although the prognosis of these injuries remains guarded, the available evidence suggests that preservation of the coronal fragment may represent a viable treatment option in carefully selected cases, provided that adequate stabilization is achieved and meticulous clinical and radiographic follow‐up is maintained.

This report has the inherent limitations of a single‐case study and a 12‐month follow‐up. Clinical and radiographic findings cannot establish the histological nature of the tissue between the fractured fragments or a specific healing pattern. In addition, the individual contribution of prolonged splinting, fragment repositioning, oral hygiene, patient age, or other treatment‐related factors to the favorable outcome cannot be determined from this case.

## 4. Conclusion

Conservative management of a cervical horizontal root fracture, including repositioning of the coronal fragment, flexible splinting, and close clinical and radiographic follow‐up, was associated with a favorable clinical and radiographic outcome at 12 months, with preservation of pulp sensibility and the natural tooth. Despite the delayed presentation, coronal fragment displacement, and cervical location of the fracture, the tooth remained clinically functional and asymptomatic at the 12‐month follow‐up. Long‐term prognosis remains to be determined through continued clinical and radiographic surveillance.

## Funding

No funding was received for this manuscript.

## Ethics Statement

Ethical approval was not required for this study because it is a single anonymized case report.

## Consent

Patient′s assent and informed consent from the patient′s legal guardian (mother) were obtained for the treatment and publication of this case report and the accompanying clinical images.

## Conflicts of Interest

The authors declare no conflicts of interest.

## Data Availability

The data that support the findings of this study are available from the corresponding author upon reasonable request.
